# Vitronectin as a Micromanager of Cell Response in Material‐Driven Fibronectin Nanonetworks

**DOI:** 10.1002/adbi.201700047

**Published:** 2017-08-10

**Authors:** Marco Cantini, Karina Gomide, Vladimira Moulisova, Cristina González‐García, Manuel Salmerón‐Sánchez

**Affiliations:** ^1^ Division of Biomedical Engineering School of Engineering University of Glasgow Oakfield Avenue G128LT Glasgow UK

**Keywords:** cell differentiation, cell response, fibronectin, protein adsorption, vitronectin

## Abstract

Surface functionalization strategies of synthetic materials for regenerative medicine applications comprise the development of microenvironments that recapitulate the physical and biochemical cues of physiological extracellular matrices. In this context, material‐driven fibronectin (FN) nanonetworks obtained from the adsorption of the protein on poly(ethyl acrylate) provide a robust system to control cell behavior, particularly to enhance differentiation. This study aims at augmenting the complexity of these fibrillar matrices by introducing vitronectin, a lower‐molecular‐weight multifunctional glycoprotein and main adhesive component of serum. A cooperative effect during co‐adsorption of the proteins is observed, as the addition of vitronectin leads to increased fibronectin adsorption, improved fibril formation, and enhanced vitronectin exposure. The mobility of the protein at the material interface increases, and this, in turn, facilitates the reorganization of the adsorbed FN by cells. Furthermore, the interplay between interface mobility and engagement of vitronectin receptors controls the level of cell fusion and the degree of cell differentiation. Ultimately, this work reveals that substrate‐induced protein interfaces resulting from the cooperative adsorption of fibronectin and vitronectin fine‐tune cell behavior, as vitronectin micromanages the local properties of the microenvironment and consequently short‐term cell response to the protein interface and higher order cellular functions such as differentiation.

## 1 Introduction

Engineering biologically active microenvironments to support tissue regeneration demands careful consideration of the protein interface that governs the interaction between synthetic materials and adhering cells. The design of this interface relies on the mimicry of the physical and biochemical cues of extracellular matrices (ECMs) that are encountered in vivo and regulate cell behavior, including phenomena like adhesion, migration, proliferation, and differentiation. Like other proteins that constitute physiological ECMs, vitronectin (VN) has a plethora of functions in tissue repair and homeostatic processes, including remodeling, and has been recently indicated as a biological “superglue.”^1^ This 75 kDa adhesive glycoprotein is a substantial component of plasma (≈300 µg mL^−1^)^2^ and is also found in the extracellular space of different tissues. Contrary to other ECM proteins with structural functions, like collagens, fibronectin (FN) or laminin, VN is a “matricellular” protein: its functions depend on the interaction with other species, including matrix proteins, cell‐surface receptors, or other molecules such as cytokines.^3^ It exhibits a high degree of conformational flexibility, and each conformation supports distinct biological activity.^4^ Ultimately, through its direct or indirect interactions with several extracellular species, VN is involved in various physiological and pathological processes, which include response to tissue trauma, angiogenesis, and cancer progression.^5^ The protein also contains an arginine–glycine–aspartic acid (RGD) motif which mediates binding to different members of the integrin family (α_v_β_1_, α_v_β_3_, α_v_β_5_, α_v_β_6_, α_v_β_8_, and α_IIb_β_3_).^6^ Engagement of VN with cell surface receptors contributes to cell adhesion and integrin‐mediated signal transduction. For example, binding of VN to its main integrin receptor (α_v_β_3_) was reported to be involved in the initiation of the assembly of FN fibrils by cells.^7^ The role of adsorbed VN during initial cell–biomaterials interactions has also been addressed in different studies,^8^ revealing that the protein undergoes dynamic remodeling at the cell–material interface.^9^ Moreover VN, together with FN, is the main adhesive component of sera used in cell culture.^10^

Fibronectin, a higher‐molecular‐weight glycoprotein found in soluble form in blood and in an insoluble form in connective tissues,^11^ is, on the other hand, an abundant structural component of the ECM. Its importance in the mediation of cell adhesion was early recognized.^12^ It is a dimer consisting of two subunits of ≈220 kDa, each containing three types of repeating units that mediate interactions with ECM proteins, several members of the integrin family, and other molecules. As a result, FN plays a crucial role in the regulation of cell survival and phenotype expression in vivo^13^ and in vitro when directly adsorbed on a variety of substrates or as a component of serum.^14^ Moreover, the engagement of FN with integrins promotes a force‐mediated reorganization of the molecule, which results in the formation of matrix fibrils through FN–FN lateral association (fibrillogenesis); the occurrence and the intensity of this phenomenon for FN adsorbed onto synthetic surfaces are considered a key factor in the biocompatibility of a material and in determining cell behavior.^15^

**Figure 1:**
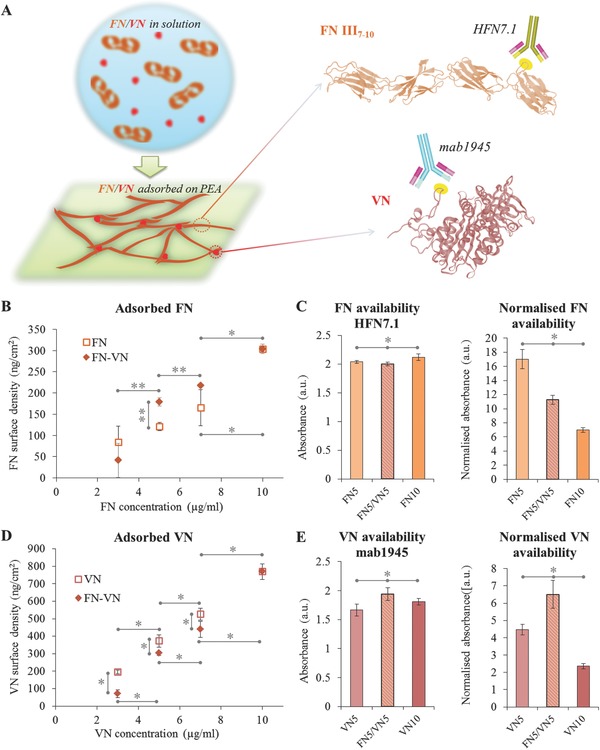
Adsorption of fibronectin and vitronectin on PEA under non‐competitive and competitive conditions. A) Schematic representation of the adsorption of FN and VN from solution onto PEA surfaces and models for the III_7–10_ domains of FN (FNIII_7–10_) and for the entire VN molecule, highlighting the RGD sequence on each protein in yellow, along with the binding sites for HFN7.1 and mab1945 antibodies; models are adapted from Protein Data Bank (PBD) ID: 1FNF^23^ for FNIII_7–10_ and PBD ID: 1SSU^24^ for VN. B) Surface density of adsorbed FN on PEA from single protein solutions (FN3 (3 µg mL^−1^), FN5 (5 µg mL^−1^), FN7 (7 µg mL^−1^), and FN10 (10 µg mL^−1^)) or mixtures with VN (FN3/VN7 (FN = 3 µg mL^−1^, VN = 7 µg mL^−1^), FN5/VN5 (FN = 5 µg mL^−1^, VN = 5 µg mL^−1^), FN7/VN3 (FN = 7 µg mL^−1^, VN = 3 µg mL^−1^)). C) Availability of the central integrin‐binding domain of FN as measured by binding of HFN7.1 monoclonal antibody on single protein and mixed proteins coatings; in the second graph, the availability is normalized by the amount of adsorbed FN. D) Surface density of adsorbed VN on PEA from single protein solutions (VN3 (3 µg mL^−1^), VN5 (5 µg mL^−1^), VN7 (7 µg mL^−1^), and VN10 (10 µg mL^−1^)) or mixtures with FN. E) Availability of the integrin‐binding domain of VN after adsorption on PEA as measured by binding of mab1945 monoclonal antibody; in the second graph, the availability is normalized by the amount of adsorbed VN. Statistically significant differences are indicated with *p < 0.05 and **p < 0.1.

Surface chemistry has been revealed, together with other surface properties, to be able to modulate the adsorption of different biologically relevant molecules, including VN and FN,^16^ regulating eventually the fate of the cells which subsequently adhere to that biointerface.^14^, ^17^ For example, we have previously shown that specific surface chemistries, e.g., poly(ethyl acrylate) (PEA), trigger the cell‐free assembly of FN into physiological‐like matrices.^18^ These FN fibrils have enhanced biological activity due to the simultaneous exposure of the central cell‐binding domain (FNIII_9–10_) that promotes α_5_β_1_ engagement and of a promiscuous growth factor (GF)‐binding domain (FNIII_12–14_) that allows for integrin‐GF synergistic signaling.^19^ This work further explores the effect of VN on material‐driven FN fibrillogenesis, by co‐adsorbing both proteins onto PEA surfaces (**Figure** **1**A).^20^ The introduction of this multifunctional adhesive glycoprotein known for its matricellular role in vivo will augment the complexity of the artificial fibrillar microenvironment. Moreover, it will shed light on the interaction between these two fundamental components of interstitial ECM and key adhesive factors of serum in a setting that mimics a physiological fibrillar microenvironment.

## 2 Results

### 2.1 Protein Adsorption

In this study, the role of VN in the modulation of the adsorption and material‐driven fibrillogenesis of FN on PEA was characterized, following previous indications that VN confers higher mobility to the protein matrix formed at the material interface and adds complexity to its biological activity.^20^ As previously reported, the presence of VN enhanced FN adsorption.^20^ Figure 1B shows the amount of FN on PEA after 1 h adsorption under noncompetitive or competitive conditions: FN was absorbed either from single protein solutions of concentrations 3, 5, 7, and 10 µg mL^−1^ (designated as FN3, FN5, FN7, and FN10) or from 10 µg mL^−1^ FN/VN mixtures in different weight ratios (30%/70%, 50%/50%, and 70%/30%, designated as FN3/VN7, FN5/VN5, and FN7/VN3). As expected, the surface density of adsorbed FN increased with the concentration of FN in the coating solution, regardless of the presence, or not, of VN (Figure 1B). Most interestingly, a higher amount of FN was adsorbed onto PEA surfaces when the proteins were competitively adsorbed from mixtures containing as much as 50% VN. Beside the amount of adsorbed FN, the protein interface was further characterized in terms of the availability of cell‐binding domains on both adsorbed FN and VN (indicated in Figure 1A). The exposure of the central binding domain on FN (containing the RGD sequence) was measured via binding of the monoclonal antibody HFN7.1, which is targeted against the flexible linker between the ninth and tenth type III repeats of the protein, and is a receptor‐mimetic probe for integrin binding and cell adhesion.^21^ Minimal differences were found in the availability of the cell‐binding domain on the adsorbed protein independently of the concentration of the protein solution (only 5 and 10 µg mL^−1^ FN solutions were used) or of the presence of VN (FN5/VN5) (Figure 1C); when these results were normalized by the amount of adsorbed FN, the exposure of the RGD domain per protein diminished with increasing FN concentration or when VN was added in the coating solution.

Similar to FN, the amount of VN adsorbed onto the surface increased with the concentration of the protein in the coating solution (Figure 1D); in this case, though, adsorption from FN/VN mixtures led to a decrease in the surface density of adsorbed VN. Furthermore, the availability of VN for cell binding was assessed using a monoclonal antibody (mab1945) known to inhibit cell adhesion to VN when added to VN‐coated substrates.^22^ On pure VN coatings, the availability of the protein increased slightly with VN concentration (Figure 1E; VN5 and VN10, indicating adsorption from solution of concentration 5 and 10 µg mL^−1^, respectively). Surprisingly, co‐adsorption of the protein with FN (FN5/VN5) led to an increase of VN exposure (higher even than VN10), despite the lower adsorbed amount (Figure 1E); normalized availability values showed that the exposure of the RGD domain per protein diminished with increasing VN concentration, while it increased when FN was also present.

**Figure 2:**
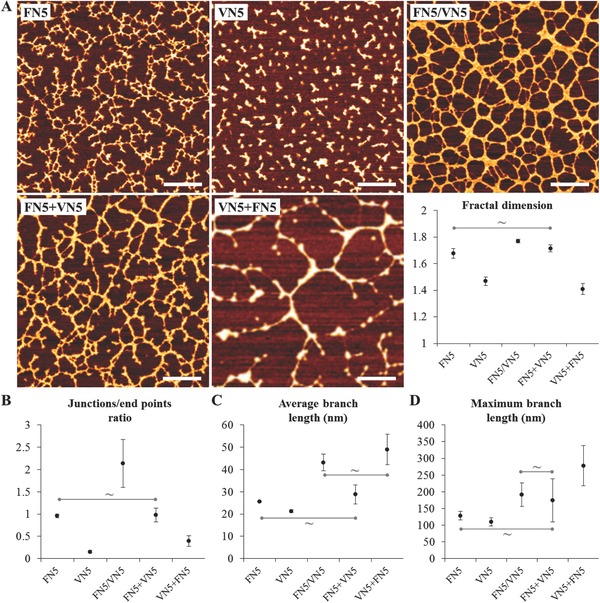
Structure and distribution of FN and VN adsorbed, co‐adsorbed, or consecutively adsorbed on PEA. A) Phase signal in tapping mode AFM: FN5, adsorption of FN (5 µg mL^−1^); VN5, adsorption of VN (5 µg mL^−1^); FN5/VN5, co‐adsorption of FN (5 µg mL^−1^), and VN (5 µg mL^−1^); FN5+VN5, consecutive adsorption of FN and VN; VN5+FN5, consecutive adsorption of VN and FN; fractal dimension of the adsorbed protein layers. B) Ratio between junctions and end points of the protein matrix. C) Average length of the branches of the protein network. D) Maximum length of the branches. All differences are statistically significant (*p* < 0.05), with the exception of the ones indicated with ∼. Scale bar: 200 nm.

Phase imaging in tapping mode AFM was then used to analyze the distribution and micro‐ and nanostructures of the adsorbed protein (**Figure** **2**); adsorption of FN on PEA promoted organization of the protein into fibrillar structures resembling FN organization in vivo or in vitro by cells (Figure 2A, FN5).^18^ On the other hand, VN was adsorbed in the form of small aggregates (Figure 2A, VN5). When both proteins were co‐adsorbed, a protein network with thicker fibrils and a higher degree of interconnectivity was obtained (Figure 2A, FN5/VN5), in agreement with previous results.^20^ This observation was confirmed by quantifying the level of complexity and connectivity of the protein matrix: addition of VN during the adsorption increased the fractal dimension and the ratio of junctions to end points (Figure 2A,B); moreover, the length of the matrix branches increased significantly from ≈25 to ≈43 nm (correspondingly, there was an increase of the maximum branch length from ≈130 to ≈190 nm) (Figure 2C,D). Further insight into the adsorption of the two proteins was gained by consecutively adsorbing VN (or FN) on a previously adsorbed FN (or VN) layer. When VN was adsorbed onto an FN‐coated polymer surface, a protein network similar to the one obtained from a pure FN solution was observed, the only difference being a slight thickening of the matrix fibrils (Figure 2A, FN5+VN5); both matrices had indeed similar fractal dimensions, junctions to end points ratio, and branch lengths. On the other hand, when FN was adsorbed onto VN‐coated PEA, large protein fibrils were observed that included most of the previously adsorbed VN aggregates (Figure 2A, VN5+FN5). Quantification of the images confirmed that precoating the surface with VN, although it diminished the connectivity of the protein layer, brought about an increase of the branch length to similar levels as the co‐adsorption.

**Figure 3:**
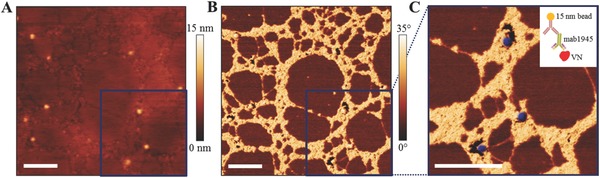
AFM imaging of immunogold labelled VN molecules on FN/VN‐coated PEA. A) Height signal in tapping mode AFM after co‐adsorption of FN (5 µg mL^−1^) and VN (5 µg mL^−1^) on PEA and immunogold staining: a secondary antibody bound to a 15 nm gold nanoparticle was used to identify available VN molecules as brighter peaks in the image. B) Corresponding phase signal. C) Magnification of the phase signal: the image is merged with the thresholded height signal (in blue) to identify the location of the gold nanoparticles. The primary antibody mab1945 recognizes VN molecules available for cell binding and the secondary antibody is tagged with a 15 nm gold nanoparticle. Scale bar: 200 nm.

To elucidate the role of both proteins within this protein matrix, VN molecules available for cell binding were tagged with a monoclonal antibody (mab1945) and then with a secondary antibody bound to a 15 nm gold nanoparticle (**Figure** **3**). VN molecules, indicated by the height signal corresponding to the presence of nanoparticles, were identified by thresholding; superposition of the phase image of the protein network with the thresholded height magnitude identified the branch points of the matrix as the preferred localization for the exposed VN molecules (Figure 3C), which have an average spacing of ≈350 nm.

### 2.2 Cell‐Mediated FN Reorganization

**Figure 4:**
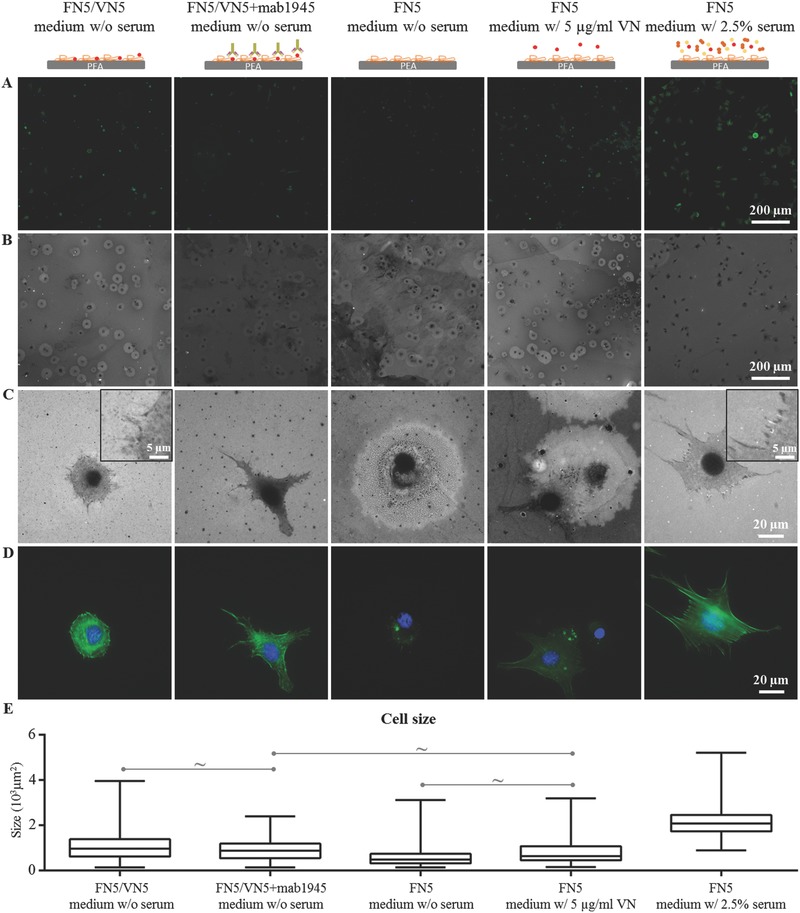
Cell‐mediated FN reorganization after 3 h incubation on FN/VN‐coated surfaces in serum‐free conditions. A) Actin cytoskeleton (green) and nuclei (blue). B) Corresponding cell‐mediated FN reorganization. C) Magnification of FN reorganization around single cells; insets indicate examples of FN reorganization by cells. D) Corresponding actin cytoskeleton (green) and nuclei (blue). E) Cell size. Mab1945 is used to block VN availability (second column); controls include reorganization of coatings of sole FN in serum‐free conditions (third column), with 5 µg mL^−1^ of VN in the culture medium (fourth column), and in the presence of 2.5% serum (fifth column). All differences are statistically significant (*p* < 0.05), with the exception of the ones indicated with ∼.

Cell‐mediated remodeling of the FN adsorbed on the surface was evaluated via immunostaining using C2C12 cells in serum‐free conditions (**Figure** **4**). When seeded on surfaces coated with 5 µg mL^−1^ of FN, most cells adopted a rounded morphology after 3 h and a brighter area in the stained FN layer appeared around the cells (Figure 4, third column). This shrunk cell morphology (resulting in a small cell size, Figure 4E) and the surrounding imprint on the protein interface indicated impaired early cell adhesion; this is attributed to the restricted nanoscale mobility of FN molecules as a consequence of high strength of interaction between FN and the underlying polymer (PEA), which leads to proteolysis.^25^ A certain degree of recovery from this effect could be observed when cells were seeded onto substrates coated with a mixture of FN and VN; many cells still left an imprint on the FN layer surrounding them, but indications of FN reorganization by cells (Figure 4C, first column) and of a more spread cell morphology (and bigger cell size, Figure 4E) were found. This positive influence of VN integration within the protein matrix on early cell response was maintained even when availability of VN for integrin binding was blocked using mab1945 antibody (Figure 4C (second column),E). As controls, cells seeded in serum‐containing culture medium were observed to spread (Figure 4E) and reorganize FN (Figure 4, fifth column); on the other hand, addition of VN to the medium did not trigger the same cell response than adding serum (Figure 4 (fourth column), E).

### 2.3 Cell Differentiation

**Figure 5:**
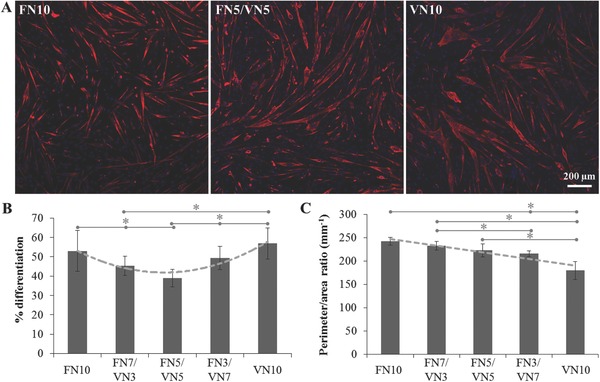
Myogenic differentiation of C2C12 cells on PEA surfaces coated with mixtures of FN and VN in different ratios. A) Sarcomeric myosin‐positive cells (red) and nuclei (blue) after 4 d of culture. B) Differentiation levels measured as the percentage of sarcomeric myosin‐positive cells. C) Perimeter‐to‐area ratio of the myotubes as an indicator of cell fusion. Dotted lines are guides for the eye. Statistically significant differences are indicated with *p < 0.05.

The effect of VN was further investigated via differentiation assays. C2C12 cells were cultured on substrates coated with FN, VN, or mixtures of the two, and the level of myogenic differentiation measured via staining of sarcomeric myosin (**Figure** **5**A). A nonmonotonic dependence of the degree of differentiation, characterized as the percentage of sarcomeric‐myosin positive cells, was observed as the content of VN increased (Figure 5B); highest differentiation levels were found on pure protein coatings (FN10 and VN10), while a minimum was reached for a 50/50 protein ratio in the coating solution. Moreover, the presence of VN triggered higher levels of cell fusion, with the formation of bigger multinucleated myotubes compared to sole FN; this was measured by the perimeter‐to‐area ratio of the myotubes, which was shown to monotonically decrease with the content of VN, as thicker myotubes were formed (Figure 5C). Results for the full series of pure protein coatings are reported in Figure S1 (Supporting Information).

**Figure 6:**
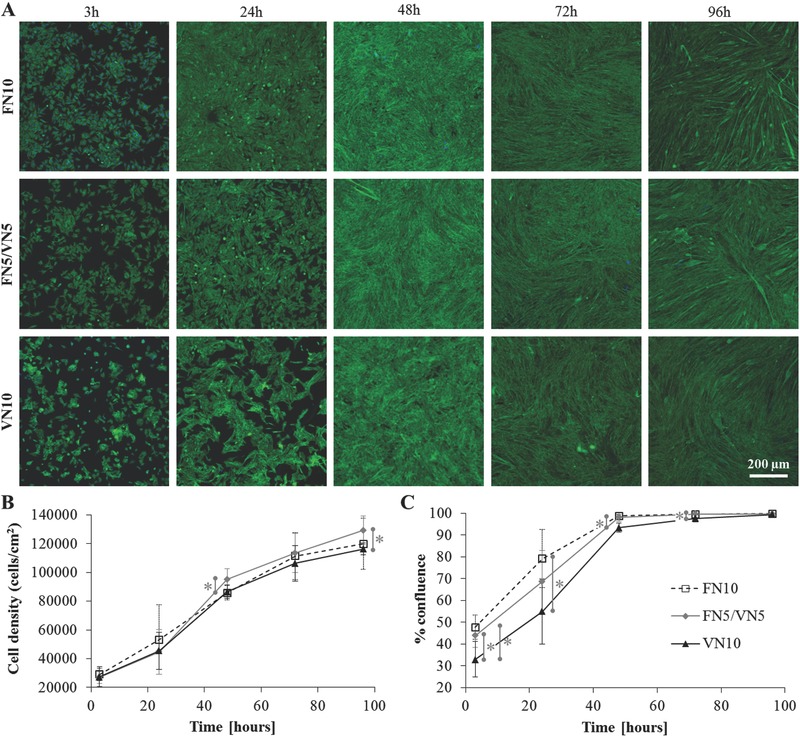
C2C12 cells differentiation over time (time points 3 h, 24 h, 48 h, 72 h, and 96 h). A) Actin cytoskeleton (green) and nuclei (blue) on single protein coatings (10 µg mL^−1^ of FN or VN) and mixed FN5/VN5 coating. B) Cell density over time. C) Cell confluence over time. Statistically significant differences are indicated with *p < 0.05.

Observation of cell differentiation over time revealed that this phenomenon is likely related to an earlier onset of cell fusion in the presence of VN (**Figure** **6**A). Indeed, even if cell proliferation followed a similar trend independently of the protein coating on the surface (Figure 6B), the presence of VN favored the organization of cells into clusters at early time points, as measured by the lower confluence degree with respect to pure FN coatings (Figure 6C).

**Figure 7:**
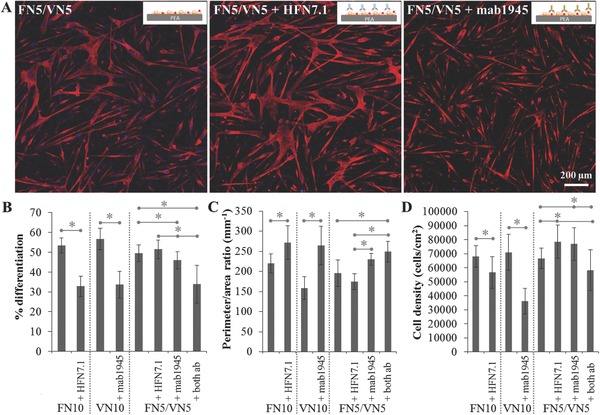
Myogenic differentiation of C2C12 cells on FN/VN‐coated PEA surfaces after blocking the RGD motif on FN or VN using monoclonal antibodies (HFN7.1 for FN, mab1945 for VN). A) Sarcomeric myosin‐positive cells (red) and nuclei (blue) after 4 d of culture. B) Differentiation levels measured as the percentage of sarcomeric myosin‐positive cells. C) Perimeter‐to‐area ratio of the myotubes as an indicator of cell fusion. D) Cell density. Controls include single protein coatings. Statistically significant differences are indicated with *p < 0.05.

Further insight into the role of the protein interface in the differentiation process was obtained by blocking the availability of the RGD sequence on either FN or VN using the monoclonal antibodies HFN7.1 and mab1945, respectively (**Figure** **7**A). Inhibiting integrin binding to the RGD motif drastically lowered the differentiation levels on pure protein coatings (from ≈55% to ≈30%; Figure 7B). This was accompanied by decreased levels of cell fusion (higher perimeter‐to‐area ratios) and reduced cell density; the effect was more pronounced on VN‐coated substrates (Figure 7C,D). On substrates coated by a 50/50 mixture of both proteins, only blocking the availability of VN led to a significant decrease of differentiation (Figure 7B); the levels were nevertheless only slightly affected. Impaired cell binding to VN resulted in hindered cell fusion and lower differentiation levels compared to blocking binding to FN through its RGD sequence.

## 3 Discussion

**Figure 8:**
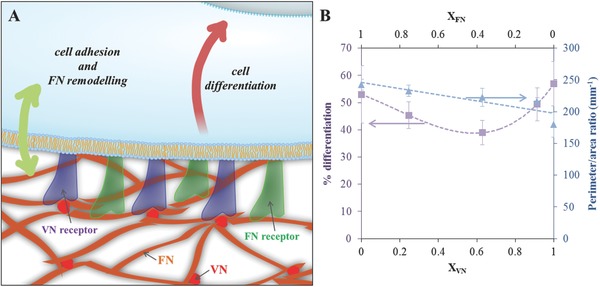
VN as a micromanager of the fibrillar FN microenvironment and of cell response. A) Matricellular VN, a smaller molecular weight protein than structural FN, is integrated in the protein network and acts a plasticizer of the FN fibrils, increasing the mobility of the protein interface; it also gains exposure for cell binding via VN receptors (α_v_ integrins). Altered physical and biological properties of the microenvironment fine‐tune cell response: cell adhesion, remodeling of the adsorbed FN, and ultimately cell differentiation. B) Cell differentiation and cell fusion (indicated by the perimeter‐to‐area ratio) as a function of the ratio of adsorbed VN (*X*_VN_) or FN (*X*_FN_) at the protein interface; dotted lines are guides for the eye.

Various studies have sought to combine the customization of material bulk and surface properties with relevant proteins to prompt the production of microenvironments that control cell response, with the ultimate objective of targeting cell differentiation, e.g., in mesenchymal stem cells, to defined lineages.[[qv: 14a,c,26]] This work builds on this concept by engineering artificial fibrillar microenvironments that exploit the interaction between one of the main structural ECM components, FN, and a particular surface chemistry, PEA. We have previously shown that this acrylate is able to trigger the cell‐free organization of FN molecules into physiological‐like nanofibrils upon adsorption of the protein onto the material surface, regardless of surface topography;^18^, ^27^ the resulting biointerface promotes cell adhesion and differentiation in vitro and supports bone regeneration in vivo.^19^, ^28^ We have also observed that other proteins or protein fragments affect this material‐driven FN assembly,^20^, ^28^ and we have identified VN as a promising candidate to alter FN organization at the cell–material interface.^20^ VN is in fact a low‐molecular‐weight matricellular component of the ECM and main adhesive component of serum, known to organize and micromanage the local hydrogel milieu in vivo through interactions with several other ECM molecules;^1^ indeed, it forms multiprotein complexes with a repertoire of biologically active species, modulating their biological functions. Here, we elucidate its role and interactions in an artificial fibrillar matrix which mimics a physiological ECM (**Figures** 1A and **8**A).

The protein/material interface was characterized by observing the amount, conformation, and organization of the adsorbed proteins following co‐adsorption of structural FN and matricellular VN in different ratios. Results interestingly reveal that addition of up to 50% of VN to the coating solution leads to cooperative effects during the adsorption process of both proteins, rather than to the competitive effects that are usually observed when proteins are absorbed from mixtures.[[qv: 16b,29]] Indeed, FN is adsorbed in higher amounts when co‐adsorbed with VN compared to adsorption of the single protein (Figure 1B), the availability of VN is enhanced despite its lower surface density (Figure 1D,E), and ultimately fibril formation and interconnection is improved (Figure 2). Several phenomena likely contribute to determine this cooperative effect, as a result of the interactions between the two adsorbing species while the structural component, FN, is undergoing a material‐induced conformation change and subsequent self‐assembly. On the one hand, VN does not seem to alter the biological properties of the adsorbed FN; after co‐adsorption, the protein still forms biologically functional fibrils (Figure 1C). Moreover, consecutive adsorption of VN on top of a preadsorbed FN layer leads to protein networks with similar fractal properties (Figure 2A). On the other hand, VN affects the physical properties of the microenvironment; the thicker and longer fibrils are likely the result of the indirect interaction of VN molecules with the adsorbing FN. VN, integrated in the protein network but without any direct binding domain with FN,[[qv: 1,5b,30]] facilitates the mobility of FN at the interface with the material surface, contributing to faster dynamics in the formation of the protein matrix.^20^ This is in accordance with previous results that show that a higher interfacial mobility is correlated with faster adsorption dynamics and FN organization into nanonetworks;[[qv: 25b,31]] here the higher interfacial mobility is obtained due to the presence of VN, a protein with lower molecular weight which plays the role of plasticizer of larger FN molecules; in Bathawab et al.[[qv: 25b]] it is translated from an increased polymer surface mobility. Other studies have also reported higher FN adsorption on surfaces with increased chain flexibility.^32^ Furthermore, VN acts as a nucleation point for the adsorption of FN molecules, as exemplified by adsorbing FN onto preadsorbed clusters of VN molecules; the resulting large protein fibrils have similar lengths to the ones achieved through co‐adsorption and include most of the VN aggregates (Figure 2). Thus, we speculate that VN acts similarly during the initial stages of adsorption from a mixture of the proteins, which are known to be dominated by the species of lower molecular weight.^33^ As part of the ensuing protein network, VN molecules appear to gain higher exposure when adsorbed onto PEA in the presence of FN compared to the aggregates formed upon adsorption from a pure VN solution (Figure 1E); interestingly, available molecules are preferentially located in the branch points of the matrix (Figure 3), pointing again at their activity as nucleation points. This enhanced and organized availability cannot be ascribed to a change in the amount of adsorbed VN (VN surface density diminishes during co‐adsorption, Figure 1D), suggesting that the protein undergoes conformational reorganization following its interaction with FN. VN is known to interact with the extracellular matrix through its collagen‐ and heparin‐binding domains, but has not been reported to interact directly with FN in vivo.^1^, ^34^ Electrostatic interactions, which have been suggested to drive FN fibrillogenesis on negatively charged material surfaces,^35^ might influence the affinity between the two adsorbing species and ultimately affect their structural organization. On PEA, FN has in fact been proposed to orient at the surface so that its hydrophobic and its positively charged domains (e.g., the heparin‐binding fragment, FNIII_12–14_) interact with the slightly negative charged surface of the polymer (negative net charge in the —COO— group of PEA);^36^ this, in turn, prompts the exposure of negatively charged domains that would otherwise be buried within the compact form of the protein (e.g., III_2–3_).^35^ The positively charged heparin‐binding domain of VN might then interact with the domains exposed by FN, allowing for a conformational transition of VN from its folded state, which aligns the amino‐terminal acidic domains containing the RGD motif with the heparin‐binding domain, into an extended form.[[qv: 5b]] Despite this lack of direct interaction, the behavior observed here is in line with reports indicating that, in vivo, the function of the protein is influenced and determined by its interactions with other species.^1^ For example, Peterson et al. showed that the interaction of VN with plasminogen activator inhibitor type 1 during hemostatic processes alters the adhesive functions of VN, enhancing its cell/matrix‐binding properties as it multimerizes and is incorporated into higher order complexes.^37^ In the artificial but physiological‐like FN‐based system used in this work, VN appears to regulate both the physical properties of the matrix (by enhancing the mobility of the protein network) and the biological properties (by enhancing its own biological activity).

Using a simple cell model with differentiation capability, C2C12 myoblasts, we explored whether this apparent regulation of the microenvironment properties translated into a control of cell response (Figure 8). It is established that cell adhesion and differentiation are enhanced on fibrillar FN compared to its globular solution conformation;^28^, ^38^ in particular, we observed that material‐driven FN fibrils are able to sustain high myogenic differentiation levels^28^ and that cell adhesion is improved with increased FN density^38^ or in the presence of VN.^20^ Here, we set out to explore short‐term cell response to the protein interface looking at cell‐mediated remodeling following cell attachment; the ability of cells to reorganize their surroundings is in fact linked to the biocompatibility of the material and its ultimate fate in vitro or in vivo.^15^ We have previously observed strong cell adhesion with traces of reorganization of the adsorbed FN by cells in the presence of serum during the culture,[[qv: 25b,31]] this is confirmed by the results in this study, as seen in Figure 4, fifth column, where good cell spreading (Figure 4E) and FN reorganization at the cell edges (Figure 4C) can be seen after 3 h of culture of C2C12 cells onto fibrillar FN on PEA. However, when cells are seeded in serum‐free conditions, adhesion is impaired, as cells are unable to reorganize the underlying layer due to the strong interaction between the FN fibrils and the underlying substrate (Figure 4, third column).^39^ This strong interaction, confirmed via atomic force spectroscopy,[[qv: 25a]] entails restricted nanoscale mobility of the protein, with cells ultimately trying degrading the interface via proteolysis.[[qv: 25a,40]] The presence of VN integrated in the FN fibrils at the material interface leads to a recovery from this effect, as cells appear to be able to spread (Figure 4E) and reorganize the adsorbed FN (Figure 4C, first column). It has been, in fact, reported that engagement of VN receptors might initiate assembly of FN by cells,^7^ and this might be in line with our observations. On the other hand, blocking the availability of VN to cells using a monoclonal antibody does not ablate cell response (Figure 4, second column), suggesting that the increased mobility granted by the VN molecules in the FN matrix is sufficient to allow for a better cell attachment and FN reorganization in serum‐free conditions. This role of VN as a micromanager of the fibrillar microenvironment is confirmed by the mixed cell response when VN is added simply as a soluble factor during the culture (Figure 4, fourth column); cells mostly fail to reorganize the underlying protein interface and adopt a shrunk morphology, as the physical properties of the microenvironment are unchanged and the engagement of VN receptors is less efficient. VN is, in fact, largely unreactive in aqueous phase.^1^

Myogenic differentiation assays interestingly revealed a nonmonotonic cell response to these FN/VN interfaces: differentiation levels are higher on pure coatings and diminish for ratios of adsorbed VN to total adsorbed protein (*X*_VN_) up to 60% (Figures 5 and 8B). The high differentiation degree of C2C12 cells on fibrillar fibronectin has been previously reported, and correlated to the ability of cells to bind to unfolded FN and exert forces;^28^ besides, the fundamental role of fibronectin in myogenesis has been further established.^41^ On the other hand, the high level of differentiation on VN is likely linked to an earlier onset of cell fusion, as shown by observing cell differentiation over time (Figure 6). The engagement of VN receptors favors the organization of cells into clusters at earlier time points, leading to higher levels of cell fusion and ultimately high differentiation. Additionally, VN has been previously reported to be able to sustain the adhesion, growth, and efficient myogenic differentiation of C2C12 cells.^42^ We postulate that the observed nonmonotonic response on the mixed coatings stems from the opposing effects on myogenic differentiation of the physical and biological changes in the biointerface properties granted by the integration of VN. The increase in mobility due to the plasticizing effect of VN appears to be dominant for VN ratios at the interface lower than 60%, when it leads to a decrease in cell differentiation levels. Indeed, we have previously observed a contractility‐dependent response of C2C12 cells to an increase in interfacial mobility, resulting in a decrease of differentiation.[[qv: 25b]] On the other hand, when the amount of VN is significant, the dominant factor controlling differentiation is the engagement of the VN receptors, allowing the increase of cell fusion to grant a higher differentiation degree. Preventing the engagement of cell receptors with the RGD sequence on either FN or VN confirms the role of each of the two proteins making up the interface. Indeed, blocking VN ablates the contribution of VN receptors engagement to cell fusion and differentiation, further reducing myogenic differentiation as the increased mobility of the FN interface remains the only factor playing a role in cell response. Blocking access to the central integrin‐binding domain on FN, on the other hand, has a lesser effect, with the enhanced engagement of VN receptors allowing a slight increase of the fusion and differentiation levels.

## 4 Conclusion

This work aims at augmenting the complexity of an artificial FN fibrillar microenvironment by using a multifunctional matricellular glycoprotein and main adhesive component of serum, VN. Similarly to VN activity in vivo, VN appears to micromanage the local properties of the microenvironment, altering both its physical and biological features. As a result, these substrate‐induced protein matrices have the potential to fine‐tune cell behavior, with VN micromanaging short‐term cell response to the protein interface and higher order cellular functions such as differentiation. In perspective, this system can be further employed to target cell response in tissue regeneration applications involving the use of bound GFs; VN has in fact the ability to bind several GFs, allowing the modulation of synergistic signaling events prompted by the FN fibrils.

## 5 Experimental Section

*Preparation of PEA Films and Protein Adsorption*: Thin films of PEA were obtained by spin coating a 4% w/v solution of bulk PEA in toluene at 2000 rpm for 30 s onto 12 mm glass coverslips (VWR) cleaned via sonication in ethanol; samples were dried in vacuo at 60 °C for 2 h before further characterization and use. The bulk PEA was previously polymerized through radical polymerization of ethyl acrylate (Sigma‐Aldrich) with 1% w/w benzoin (Sigma‐Aldrich) as a photoinitiator for 12 h.

Samples were sterilized via UV for 20 min and coated with the proteins at a total concentration of 10 µg mL^−1^ in Dulbecco's phosphate‐buffered saline containing calcium and magnesium (DPBS, Gibco) for 1 h at room temperature (RT). After coating, samples were rinsed once with DPBS prior to use. FN and VN, both from human plasma (Sigma‐Aldrich), were used for the coatings in different FN/VN weight (molar) ratios: 100/0 (100/0), 70/30 (28/72), 50/50 (15/85), 30/70 (7/93), and 0/100 (0/100). Pure protein coatings were also performed at different concentrations (10, 7, 5, and 3 µg mL^−1^), and consecutive FN + VN and VN + FN coatings were performed using 5 µg mL^−1^ solutions of the pure protein; samples were rinsed with DPBS after each coating. For observation via atomic force microscopy (AFM), samples were also rinsed with Milli‐Q water before drying gently with a nitrogen flow.

*Quantification of Protein Adsorption*: The amount of adsorbed FN or VN was measured via depletion of the protein from the coating solution. Specifically, the amount of FN remaining in the supernatant after adsorption for 1 h was quantified via western blot as explained in Rico et al.^43^ The amount of VN remaining in the supernatant after 1 h adsorption was quantified via enzyme‐linked immunosorbent assay (ELISA) (Human Vitronectin ELISA kit, ThermoFisher Scientific), following manufacturer instructions; the amount of protein in the coating solutions was confirmed via bicinchoninic acid assay (Micro BCA, ThermoFisher Scientific).

Availability of FN or VN for cell binding through the RGD domain was measured via a direct ELISA with monoclonal antibodies at RT. Samples were incubated with the primary antibody at 0.1 µg mL^−1^ in 1% w/v BSA/DPBS for 1 h after blocking for 30 min with 1% w/v bovine serum albumin (BSA)/DPBS; mouse HFN7.1 (DSHB) monoclonal antibody was used to detect the central integrin‐binding domain on human FN, while mouse mab1945 (Millipore) was used for human VN. Samples were then washed twice in 0.5% v/v Tween20/DPBS, and incubated with a goat antimouse HRP‐conjugated secondary antibody (Invitrogen) at 0.1 µg mL^−1^ in 1% w/v BSA/DPBS for 1 h. After washing the samples twice with 0.5% v/v Tween20/DPBS and transferring them to a clean 24‐well multiwell plate, they were incubated with an horseradish peroxidase (HRP)‐substrate solution (R&D Systems) for 20 min; the reaction was stopped using a stop solution (R&D Systems) before transferring the solution to a 96‐well multiwell plate. Absorbance was read in a Tecan plate reader at 450 nm with wavelength correction at 570 nm; three readings were performed for each sample.

*Atomic Force Microscopy*: Phase and height images were obtained for protein‐coated PEA surfaces using AFM in AC mode (Nanowizard 3 Bioscience AFM, JPK). A pyramidal silicon nitride tip, with a cantilever spring constant of ≈3 N m^−1^ and a resonance frequency of ≈75 kHz (MPP‐21120, Bruker), was used.

The phase images were processed using the JPK Data Processing software (version 5.0.84) and analyzed using the ImageJ software (1.47v). The fractal dimension was determined using the ImageJ fractal box count analysis tool, using box sizes of 2, 3, 4, 6, 8, 12, 16, 32, and 64 pixels onto 1 × 1 µm^2^ images after conversion to 8 bit grayscale, followed by thresholding and binarization. The junctions to end points ratios, and the average and maximum branch lengths were calculated by using the Analyze Skeleton ImageJ plug‐in on the skeletonized binary images.

For immunogold labeling and imaging of available VN molecules, protein‐coated samples were fixed in 4% v/v formaldehyde in DPBS for 30 min, followed by incubation with mab1945 at 2.5 µg mL^−1^ in DPBS for 1 h. After washing thrice with 0.5% v/v Tween20/DPBS for 5 min with agitation, samples were incubated with a goat antimouse secondary antibody tagged with a 15 nm gold nanoparticle (Aurion) at a 1:20 dilution in DPBS for 1 h. Samples were then washed thrice with 0.5% v/v Tween20/DPBS for 5 min with agitation, washed twice for 5 min in DPBS with agitation, fixed with 4% v/v formaldehyde in DPBS for 5 min, washed twice with Milli‐Q water for 5 min, and finally gently dried with nitrogen flow before imaging in AC mode as previously detailed. Height images were thresholded to identify the gold nanoparticles using the Gwyddion software (version 2.36) with a 60% height threshold. Thresholded height images were then superimposed to the corresponding phase signal to identify the location of the gold nanoparticles within the protein coating.

*Cell Culture*: C2C12 mouse myoblast cells were obtained from ATTC and passaged according to standard procedures using Dulbecco's Modified Eagle's Medium (DMEM, Gibco) with 4.5 g L^−1^ of d‐glucose and l‐glutamine supplemented with 1% penicillin/streptomycin (P/S, Gibco), and 20% fetal bovine serum (FBS, Gibco) as growth medium. All cells used for experiments were of passage below 8.

For FN reorganization experiments cells were seeded on the protein‐coated surfaces (50/50 mixed coatings and pure 5 µg mL^−1^ FN coatings) at a density of 5000 cells cm^−2^ in serum‐free medium. VN availability on the mixed coating was blocked by incubation of the protein coating with the mab1945 antibody at 25 µg mL^−1^ in DPBS (1:1 molar ratio to VN)^22^ followed by rinsing thrice with DPBS prior to cell culture. Controls included cells seeded onto pure FN coatings in the presence of 5 µg mL^−1^ of VN in the culture medium or in the presence of 2.5% FBS (which contains VN at roughly the same concentration). After 3 h, samples were fixed with 4% v/v formaldehyde in DPBS for 30 min at 4 °C and stained for actin, nuclei, and FN. Specifically, samples were permeabilized using a 10.3% w/v sucrose, 0.292% w/v NaCl, 0.06% w/v MgCl_2_, 0.476% w/v 4‐(2‐hydroxyethyl)‐1‐piperazineethanesulfonic acid (HEPES), 0.5% v/v Triton X‐100 solution in Milli‐Q water (pH 7.2) at RT for 5 min, blocked with 1% w/v BSA/DPBS for 30 min, and incubated with a rabbit polyclonal antibody against human FN (Sigma) diluted 1:400 in 1% w/v BSA/DPBS for 1 h. After washing twice with 0.5% v/v Tween20/DPBS, samples were incubated with a 1:200 goat antirabbit Cy3‐conjugated secondary antibody (Jackson ImmunoResearch) and a 1:100 BODIPY FL phallacidin (to stain the actin cytoskeleton; Life Technologies) solution in 1% w/v BSA/DPBS. Samples were finally mounted in a Vectashield mounting medium containing 4′,6‐diamino‐2‐phenylindole (DAPI, Vector Laboratories) to stain the nuclei and observed using an AxioObserver Z1 inverted fluorescence microscope (Zeiss). Cell size was measured from the actin cytoskeleton images using ImageJ; experiments were performed in triplicate, and at least 50 cells were measured per each condition.

For myogenic differentiation experiments, cells were seeded on the protein‐coated surfaces at a density of 20 000 cells cm^−2^ in differentiation medium (DMEM + 1% P/S + 1% insulin‐transferrin‐selenium‐ethanolamine, ITS‐X, Life Technologies) and cultured for 4 d, with the medium being replaced 3 h and 2 d after seeding. FN or VN availability was blocked by incubation of the protein coating with HFN7.1 (7.3 µg mL^−1^, 1:1 molar ratio to FN) or mab1945 (25 µg mL^−1^, 1:1 molar ratio to VN)^22^ antibody, respectively, followed by rinsing thrice with DPBS prior to cell culture. After 4 d of culture, cells were fixed and permeabilized with a 20:2:1 70% ethanol/37% formaldehyde/acetic acid solution for 10 min at 4 °C. Myogenic differentiation was then evaluated by staining for sarcomeric myosin. Samples were incubated for 1 h in a 5% goat serum/DPBS blocking buffer, followed by washing thrice with DPBS and incubating 1 h with MF‐20 mouse antibody (DSHB) at 37 °C. After washing thrice in DPBS and blocking 10 min with blocking buffer, samples were incubated with antimouse Cy3‐conjugated secondary antibody (Jackson ImmunoResearch) for 1 h, washed and finally mounted using Vectashield containing DAPI. Samples were observed using an AxioObserver Z1 inverted fluorescence microscope. The degree of myogenic differentiation was determined as the percentage of cells positive for sarcomeric myosin using the CellC cell counting software;^44^ cell density was determined by counting the total number of nuclei using the same software; experiments were performed in triplicate and at least nine 1565 × 1568 µm^2^ images were analyzed per each condition. The perimeter‐to‐area ratio of the myotubes, used as an indicator of the level of cell fusion, was calculated using ImageJ by outlining the thresholded binarized images of the sarcomeric myosin staining; at least five 1565 × 1568 µm^2^ images were analyzed per each condition. In the case of the experiments to assess cell differentiation over time, samples were fixed after 3 h, 1 d, 2 d, 3 d, and 4 d of culture using 4% v/v formaldehyde in DPBS for 30 min at 4 °C. Then, samples were permeabilized using a 10.3% w/v sucrose, 0.292% w/v NaCl, 0.06% w/v MgCl_2_, 0.476% w/v HEPES, 0.5% v/v Triton X‐100 solution in Milli‐Q water (pH 7.2) at RT for 5 min, blocked with 1% w/v BSA/DPBS for 30 min and incubated with a 1:100 BODIPY FL phallacidin solution in 1% w/v BSA/DPBS to stain the actin cytoskeleton. Samples were finally mounted in a Vectashield mounting medium containing DAPI to stain the nuclei and observed using an AxioObserver Z1 inverted fluorescence microscope. Cell density was measured using CellC to count the total number of cell nuclei per image, while the percentage of confluence was determined from the thresholded binarized actin staining images using ImageJ; experiments were performed in triplicate and at least six 1565 × 1568 µm^2^ images were analyzed per each condition.

*Statistical Analysis*: Experiments were performed at least in triplicate, and the values of the measurements are reported as the average of the samples, with the variability expressed in terms of standard deviation. Statistically significant differences were determined by one‐way analysis of variance (ANOVA), with *p* < 0.05.

## Conflict of Interest

The authors declare no conflict of interest.

## Supporting information

Supplementary
