# Supplementary material for: Vitronectin as a Micromanager of Cell Response in Material‐Driven Fibronectin Nanonetworks
Source: Adv Biosyst. 2017 Aug 10;1(9):1700047. doi: 10.1002/adbi.201700047 (PMC5822048; doi:10.1002/adbi.201700047)
Supplement: Supplementary file 1 — Supplementary [file ADBI-1-na-s001.pdf]

Copyright WILEY-VCH Verlag GmbH & Co. KGaA, 69469 Weinheim, Germany, 2017.

ADVANCED BIOSYSTEMS

Supporting Information

for *Adv. Biosys.*, DOI: 10.1002/adbi.201700047

Vitronectin as a Micromanager of Cell Response in Material-Driven Fibronectin Nanonetworks

Marco Cantini, Karina Gomide, Vladimira Moulisova, Cristina González-García, and Manuel Salmerón-Sánchez**

Copyright WILEY-VCH Verlag GmbH & Co. KGaA, 69469 Weinheim, Germany, 2016.

Supporting Information

Vitronectin as a Micromanager of Cell Response in Material-driven Fibronectin Nanonetworks

Marco Cantini, Karina Gomide, Vladimira Moulisova, Cristina González-García and Manuel Salmerón-Sánchez**

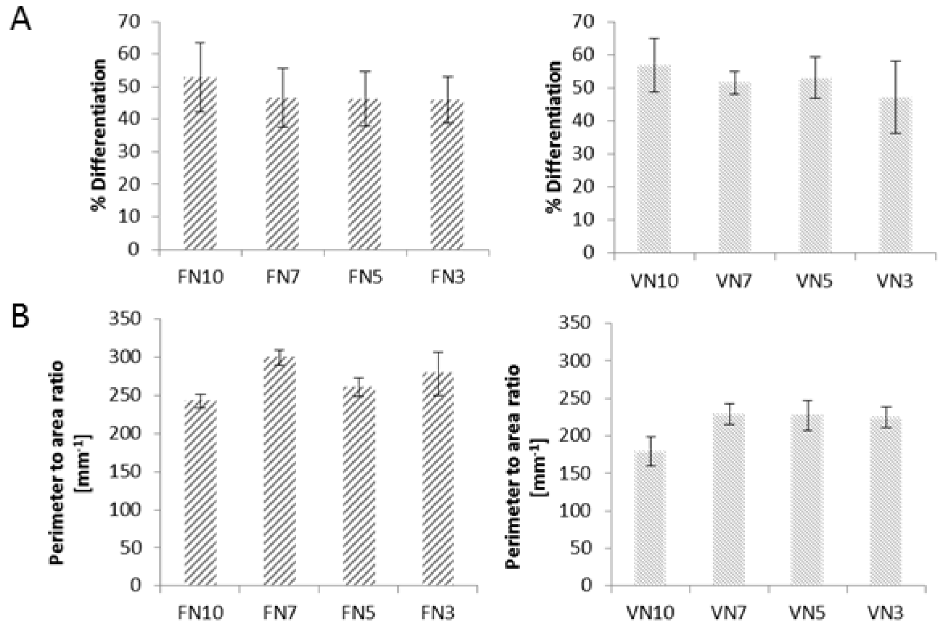

Figure S1. Myogenic differentiation of C2C12 cells on PEA surfaces coated with different concentrations of FN or VN. (**A**) Differentiation levels measured as the percentage of sarcomeric myosin-positive cells. (**B**) Perimeter-to-area ratio of the myotubes as an indicator of cell fusion. Protein concentrations: 10 μg/ml, 7 μg/ml, 5μg/ml, 3 μg/ml in DPBS.
